# ﻿A new species of *Racelda* Signoret, with taxonomical notes and a key to the males of the genus (Hemiptera, Reduviidae, Ectrichodiinae, Ectrichodiini)

**DOI:** 10.3897/zookeys.1122.84424

**Published:** 2022-09-23

**Authors:** Hélcio R. Gil-Santana, Jader Oliveira

**Affiliations:** 1 Laboratório de Diptera, Instituto Oswaldo Cruz, Av. Brasil, 4365, 21040-360, Rio de Janeiro, RJ, Brazil Instituto Oswaldo Cruz Rio de Janeiro Brazil; 2 Universidade de São Paulo, Faculdade de Saúde Pública, Laboratório de Entomologia em Saúde Pública, São Paulo, SP, Brazil Universidade de São Paulo São Paulo Brazil; 3 Laboratório de Parasitologia, Universidade Estadual Paulista “Julio de Mesquita Filho”, Faculdade de Ciências Farmacêuticas UNESP/FCFAR, Rodovia Araraquara Jaú, KM 1, 14801-902, Araraquara, SP, Brazil Universidade Estadual Paulista “Julio de Mesquita Filho” Araraquara Brazil

**Keywords:** Heteroptera, male genitalia, Neotropics, *Pseudoracelda*, *Raceldaaberlenci*, *Raceldarobusta*

## Abstract

*Raceldaottoi* Oliveira & Gil-Santana, **sp. nov.**, belonging to the tribe Ectrichodiini in the subfamily Ectrichodiinae, is described based on males from northeastern Brazil. Photographs of the male types of *Raceldaalternans* Signoret, 1863, *R.moerens* Breddin, 1898, and *R.spurca* (Stål, 1860) are presented. A summary of and notes on the taxonomic history of the genus and a key to males are provided.

## ﻿Introduction

The subfamily Ectrichodiinae in the New World includes 24 genera and more than 100 described species (Gil-Santana and Baena 2009; Gil-Santana et al. 2013, 2015, 2020; Gil-Santana 2014, 2015, 2019, 2020a, 2020b; Forthman and Weirauch 2017; Schuh and Weirauch 2020; Forthman and Gil-Santana 2021). A summary of the taxonomy of New World Ectrichodiinae was also provided by Gil-Santana et al. (2015). Forthman and Weirauch (2017) created two new tribes, Ectrichodiini and Tribelocodiini, resulting in a new composition of Ectrichodiinae (*sensu novum*) in the New World. Ectrichodiini now includes all the genera formerly belonging to Ectrichodiinae, except *Ectrichodiella* Fracker & Bruner, 1924 which has been transferred to Tribelocodiini. The latter also includes *Tribelocodia* Weirauch, 2010 (which previously belonged to Tribelocephalinae, now a junior synonym of Ectrichodiinae). An updated key to the genera of this group considering this new arrangement of Ectrichodiinae was presented by Forthman and Gil-Santana (2021).

Because of the lack of consensus of previous authors about the validity or recognition of some genera of Ectrichodiini (Dougherty 1995; Carpintero and Maldonado 1996), there is a need of a taxonomic revision and a phylogenetic analysis of all these genera (Gil-Santana et al. 2015, 2020). For instance, *Pseudoracelda* Carpintero, 1980 was considered to be a junior synonym of *Racelda* Signoret, 1863 by Dougherty (1995) but a valid genus by Carpintero and Maldonado (1996), Forero (2004), Gil-Santana et al. (2013, 2015, 2020), and Forthman and Gil-Santana (2021). It is noteworthy that in this case, Dougherty (1995: 175) justified her synonymization of these genera by stating that *Pseudoracelda* would have been erected by Carpintero (1980) for a female “whose character description fits well within the diversity seen in the sexual dimorphism of the genus *Racelda*.” She also stated that *P.macrocephala* Carpintero, 1980 “is retained as a species of *Racelda* because the unique specimen is unavailable for study.” In Ectrichodiinae, the sexual dimorphism ranges from slight (e.g., body size, development of the hemelytron, and eye and ocellar size) to extreme, where females exhibit brachyptery to aptery in both pairs of wings and major modifications in other parts of the body (Forthman and Weirauch 2017). Sexual dimorphism in *Racelda* is regarded as being strongly developed (Dougherty 1995) or extreme (Forthman and Weirauch 2017). The females are apterous, their external structures are so strongly modified that it is impossible to associate females to a species, except if associated with the respective males (Dougherty 1995). In *Racelda*, the apterous females have small eyes, the ocellar tubercle obsolete, and ocelli lacking; the posterior lobe of pronotum and the scutellum may be atrophied (Carpintero and Maldonado 1996). *Pseudoracelda*, however, was described based on a male holotype and, more importantly, also on a winged female (“allotype female”) with well-developed ocelli (Carpintero 1980), what justifies considering it valid, until a taxonomic revision and a phylogenetic analysis of all the Ectrichodiinae (or at least Ectrichodiini) genera are produced (Gil-Santana et al. 2015).

Currently, *Racelda* has six species (Carpintero and Maldonado 1996; Bérenger and Gil-Santana 2005; Gil-Santana et al. 2015).

## ﻿Materials and methods

Photographs of paratypes of *Raceldaottoi* sp. nov. (Figs 12, 16–21, 25) were taken by the junior author (JO) using a stereoscope microscope (Leica 205A) with a digital camera.

Photographs of the male syntype (Figs 3–5) and other potential (?) male syntype (Figs 6–8) of *Raceldaalternans* Signoret, 1863, deposited in the Natural History Museum, Vienna, Austria (**NHMW**), were taken by Harald Bruckner and provided by him and Herbert Zettel. Images of the male syntype of *Raceldaspurca* Stål, 1860 (Figs 58–60), deposited in the Swedish Museum of Natural History, Stockholm, Sweden (**NHRS**) (freely accessible at: http://www2.nrm.se/en/het_nrm/s/racelda_spurca.html) were provided by Gunvi Lindberg.

**Figures 1–10.**
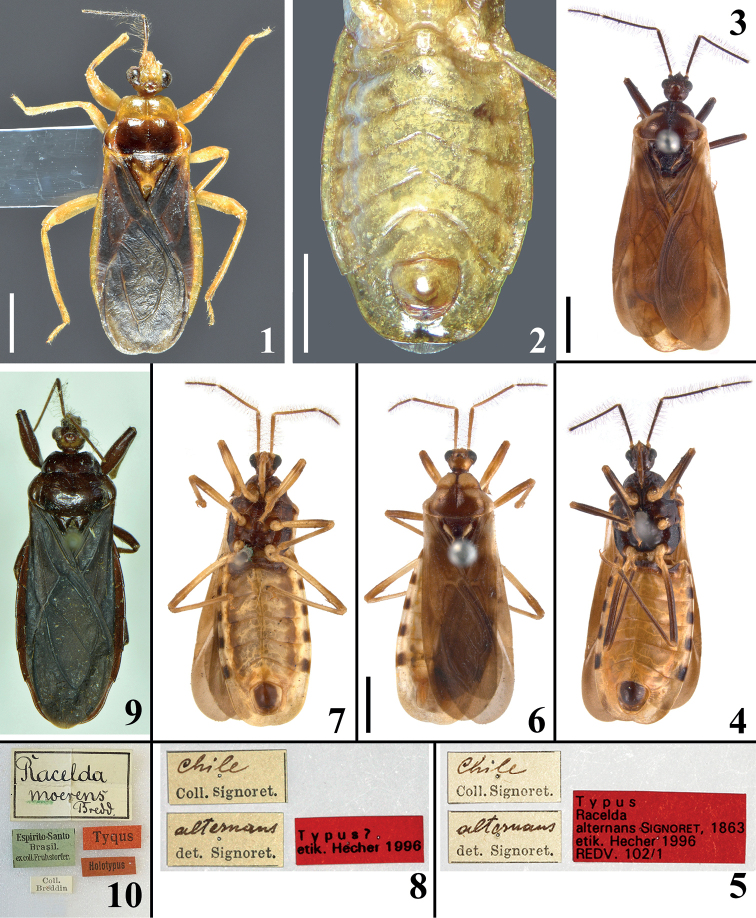
**1, 2***Raceldaaberlenci* Bérenger & Gil-Santana, 2005, male from French Guiana. **1** dorsal view **2** abdomen, ventral view. **3–8***Raceldaalternans* Signoret, 1863, male specimens deposited in NHMW. **3–5** syntype **3** dorsal view **4** ventral view **5** labels **6–8** potential syntype **6** dorsal view **7** ventral view **8** labels. **9, 10***Raceldamoerens* Breddin, 1898, male holotype deposited in SDEI**9** dorsal view **10** labels. Scale bars: 2.0 mm (**2, 3, 6**); 1.0 mm (**1**).

**Figures 11–17.**
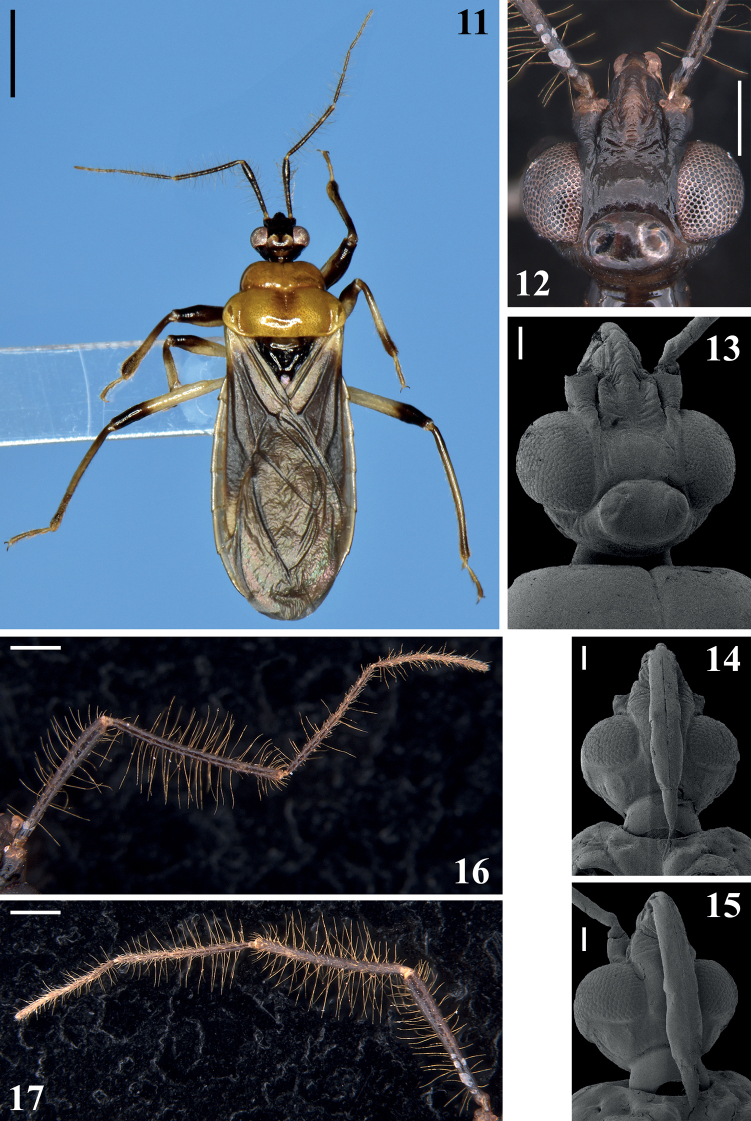
*Raceldaottoi* Oliveira & Gil-Santana, sp. nov. **11** holotype, dorsal view **12–15** head **12, 13** dorsal view **14** ventral view **15** ventrolateral view **16, 17** antenna, dorsal view **16** right **17** left. Scale bars: 2.0 mm (**11**); 0.5 mm (**12, 16, 17**); 0.2 mm (**13–15**).

The holotype and paratypes of *R.ottoi* sp. nov. (Figs 11, 39); holotype of *Raceldamoerens* Breddin, 1898 (Figs 9, 10), and non-type specimens of *Raceldaaberlenci* Bérenger & Gil-Santana, 2005 (Figs 1, 2), *R.robusta* Bérenger & Gil-Santana, 2005 (Figs 56, 57), and *R.spurca* (Figs 61, 62) were directly examined and imaged by the first author (HRG-S). Photographs were taken using digital cameras (Nikon D5200 or D5600 with a Nikon 105 mm macro lens). The respective types, depositories, and curators, who kindly allowed examining specimens, are as follows: type specimens of *R.alternans*: **NHMW**, Herbert Zettel; male holotype of *R.moerens* Breddin, 1898: Senckenberg Deutsches Entomologisches Institut, Müncheberg, Germany (**SDEI**), Stephan M. Blank.

Scanning electron microscopy images (Figs 13–15, 22–24, 26–38, 40–42, 45, 46) were obtained by the second author (JO). Two male paratypes of *R.ottoi* sp. nov. were cleaned in an ultrasound machine. Subsequently, the samples were dehydrated in alcohol, dried in an incubator at 45 °C for 20 min, and fixed in small aluminum cylinders with transparent glaze. Sputtering metallization was then performed on the samples for 2 min at 10 mA in an Edwards sputter coater. After this process, the samples were studied and photographed using a high-resolution field emission gun scanning electron microscope (SEM; JEOL, JSM-6610LV), similarly as described by Rosa et al. (2010, 2014).

The figure of the abdominal segment VIII (Fig. 43) and most figures of the male genitalia of *R.ottoi* sp. nov. (Figs 44, 47–55) were produced by the first author (HRG-S). Dissections of the male genitalia were made by first removing the pygophore from the abdomen with a pair of forceps and then clearing it in 20% NaOH solution for 24 h. The dissected structures were studied and photographed in glycerol using digital cameras (Sony DSC-W570 and DSC-W830). Drawings were made using a camera lucida. Images were edited using Adobe Photoshop CS6.

Observations were made using a stereoscope microscope (Zeiss Stemi) and a compound microscope (Leica CME). Measurements were made using a micrometer eyepiece. General morphological terminology mainly follows Schuh and Weirauch (2020). The (visible) segments of labium are numbered as II to IV, given that the first segment is lost or fused to the head capsule in Reduviidae (Weirauch 2008). In case of terms applied particularly to the Ectrichodiinae, the terminology of general morphology follows Dougherty (1995) and Forthman et al. (2016). In general, to genitalia terms, Forthman et al. (2016) are followed.

All type specimens of *Raceldaottoi* sp. nov. were collected by members of the team of the “Diversity and conservation of Hemiptera (Insecta) from the Caatinga” Project, funded by the Brazilian “Conselho Nacional de Desenvolvimento Científico e Tecnológico”, process 421413/2017-4, and authorized through the Biodiversity Authorization and Information System (SISBIO), collection permit number 62159.

The type specimens of *Raceldaottoi* sp. nov. will be deposited as follows: male holotype, 2 male paratypes in the “Coleção Zoológica do Maranhão” (**CZMA**) of the “Centro de Estudos Superiores da Universidade Estadual do Maranhão”, Caxias, Maranhão, Brazil; 1 male paratype in the “Coleção Entomológica do Instituto Oswaldo Cruz” (**CEIOC**), Rio de Janeiro, Brazil, and 2 male paratypes used to obtain SEM images will be deposited in the Dr Jose Maria Soares Barata Triatominae Collection (**CTJMSB**) of the São Paulo State University, Julio de Mesquita Filho, School of Pharmaceutical Sciences, Araraquara, São Paulo, Brazil. Additional non-type specimens of other species were or will be deposited in the Entomological Collection of the Museu Nacional da Universidade Federal do Rio de Janeiro, Rio de Janeiro, Brazil (**MNRJ**).

When describing label data, a slash (/) separates the lines and a double slash (//) different labels, and comments or translations to English of the label data are provided in square brackets ([]). All measurements are in millimeters (mm).

## ﻿Results

### ﻿Taxonomy

**Subfamily Ectrichodiinae**

#### Genus *Racelda* Signoret, 1863

Signoret (1863) created *Racelda* for the species he was describing, *R.alternans* Signoret, 1863, providing a short description of the genus. Dougherty (1995) and Carpintero and Maldonado (1996) provided redescriptions of *Racelda*. Dougherty (1995) stated that the pronotum of *Racelda* has the mid-longitudinal furrow well developed anteriorly and obsolete posteriorly, and the anterolateral corners are squared. Carpintero and Maldonado (1996) stated that the longitudinal sulcus extends along both lobes of pronotum, the circular spiracles and the short prongs on the triangular scutellum would be diagnostic of males of the genus. Forero (2004) considered the main diagnostic characteristics of *Racelda* to be: the first visible labial segment longer than the second, longitudinal sulcus of pronotum continuous on the two lobes, prolongations of the scutellum short, and strong sexual dimorphism.

With the exception of *Raceldamonstrosa* Carpintero, 1980, described based only on the female holotype, all other species of *Racelda*, *R.aberlenci* Bérenger & Gil-Santana, 2005, *R.alternans*, *R.moerens* Breddin, 1898, *R.robusta* Bérenger & Gil-Santana, 2005, and *R.spurca* (Stål, 1860) were described based only on male specimens (Signoret 1863; Stål 1860; Breddin 1898; Carpintero 1980; Bérenger and Gil-Santana 2005). There are no formal descriptions of the females of any of these species in the literature, only the figures of the dorsal habitus of the females of *R.alternans* (Carpintero and Maldonado 1996; Melo and Faúndez 2015) and *R.spurca* (Forthman and Weirauch 2017) exist. Because of that, the comments about the characteristics of the species, most of which with their females unknown or not well characterized, are based or focused only on the respective males.

##### ﻿ Racelda aberlenci

Taxon classificationAnimaliaHemipteraReduviidae

Bérenger & Gil-Santana, 2005

651D26C8-E10F-5840-B6C3-ED05DB133A8B

Figs 1 , 2

###### Material examined.

French Guiana, Bélizon, vii.2001, H. Gaspard leg., 2 males (MNRJ).

*Raceldaaberlenci* was described based on males from French Guiana and Brazil (Amazonian region) (Bérenger and Gil-Santana 2005).

##### ﻿ Racelda alternans

Taxon classificationAnimaliaHemipteraReduviidae

Signoret, 1863

CF18BE5B-2CA6-5F3F-96F4-278D383F4974

Figs 3–8

###### Type material examined.

*Raceldaalternans*. **Male syntype**: *alternans* [handwritten] / det. Signoret. [printed] // Chile [handwritten] / Coll. Signoret. [printed] // [printed red label]: Typus / Racelda /alternans Signoret, 1863 / etik. Hecher 1996 / REDV. 102/1; Male [potential] syntype: *alternans* [handwritten] / det. Signoret. [printed] // Chile [handwritten] / Coll. Signoret. [printed] // [printed red label]: Typus? / etik. Hecher 1996 (NHMW).

*Raceldaalternans* was described from Chile (Signoret 1863), and it is the type species of *Racelda* by monotypy (Maldonado 1990). It was also recorded from Argentina by Dougherty (1995).

A male syntype is deposited in NHMW (Figs 3–5). Besides this syntype, another male deposited in the same collection and with same data is considered as a potential (probable) or doubtful (“?”) syntype of *R.alternans* (Sehnal 2000) (Figs 6–8). The position of uncertainty of Sehnal (2000) about this potential syntype was based mainly on the supposed divergence between the coloration of the pronotum of this specimen and the original description of *R.alternans* by Signoret (1863). His description stated the following: prothorax [pronotum] brownish with two large lateral yellow markings, which meet almost at level of anterior furrow. This description seems to be in accordance with both specimens, possibly, even more with that which was considered as a doubtful syntype by Sehnal (2000) (Fig. 6). In the recognized syntype there are only lateral pale markings on hind lobe of pronotum which are far from meeting at the level of the transverse furrow (Fig. 3), while in the doubtful syntype, besides similar lateral pale markings on hind lobe, the fore lobe is almost completely pale, a coloration only interrupted by the mid-longitudinal furrow (Fig. 6). It is noteworthy that the drawing of the habitus (dorsal view) of *R.alternans* which accompanies the original description (“fig. 6”) clearly shows a pair of pale rounded markings on fore lobe of pronotum, although smaller than what is seen in the “?” syntype, in which almost all the fore lobe (except the median sulcus) is pale (Fig. 6). It is possible that more than these two type specimens existed when Signoret described the species, but the figure makes it clear that another specimen, with pale portions on the fore lobe of the pronotum, which are not observed in the syntype recognized by Sehnal (2000), was drawn and therefore considered by Signoret (1863) as belonging to this species. On the other hand, it is noteworthy that the quality of details and precision of the drawings in Signoret’s paper are not those required today for a scientific drawing of a specimen. Thus, it is possible that the pale markings of the pronotum had not been well depicted and the pale markings were represented smaller than they are in the “?” syntype. The lack of precision in the mentioned drawing is suggested by the pattern of the lateral pale markings of the hind lobe, which were drawn as a pair of parallel lines meeting at their apices, while in reality they are continuous markings at each side of hind lobe. Therefore, this evidence and the fact that the original labels of both type specimens are very similar and with the same handwriting (Figs 5, 8), suggest that both specimens should be considered and possibly recognized as syntypes of *R.alternans*.

Melo and Faúndez (2015) argued that *R.alternans* could be easily distinguished by the characteristic coloration of the thorax of the males. The pronotum of the male photographed by them (their fig. 11) presented a general dark coloration with a relatively large median pale marking on each side of fore lobe and the hind lobe with lateral pale markings similar to those of the syntype considered as doubtful by Sehnal (2000) (Fig. 6).

##### ﻿ Racelda moerens

Taxon classificationAnimaliaHemipteraReduviidae

Breddin, 1898

16D1157A-4FB3-5693-B0DD-C14EB401D84A

Figs 9 , 10

###### Type material examined.

**Male holotype**: *Racelda* / *moerens* [letters “*mo*” underlined with green] / Bredd. [handwritten label] // Espirito-Santo / Brasil. / ex coll. Fruhstorfer. [printed green label] // Tyqus [*sic*] [printed red label] // Holotypus [printed red label] // Coll. / Breddin [printed label] (SDEI).

According with the original description (Breddin 1898), *R.moerens* was described based on a unique male from state of Espírito Santo, Brazil (Breddin 1898). It is deposited in SDEI and bears two red labels, one reading “Tyqus” [certainly meaning “Typus”, a mere typo] and other “Holotypus” (Figs 9, 10). Gaedike (1971) listed this type specimen as the holotype of the species. It is known that several type specimens described in that time currently bear “typus”, holotypus” or “paratypus” labels, although these were not attached by the author of the species, but subsequently in curatorial practice. In any case, taking into account that the evidence supports that Breddin’s description was based on this single specimen, even not stated as the holotype in the original publication, it is considered fixed as such by monotypy (ICZN, Art. 73.1.2) and also in accordance with Gaedike (1971).

Dougherty (1995) recorded a large variation in the size of males of *R.moerens*, with a range in total length from 9.5 to 18.2 mm, although the individuals were otherwise identical. The collecting of variously sized individuals on the same date and at the same locality implies that they belonged to the same population (Dougherty 1995).

*Raceldamoerens* has been recorded so far only from Brazil (Maldonado 1990; Dougherty 1995).

##### ﻿ Racelda ottoi

Taxon classificationAnimaliaHemipteraReduviidae

Oliveira & Gil-Santana sp. nov.

640E7BDE-C8F8-50F6-B21E-91749136FEB6

http://zoobank.org/57A592A5-8D05-4EAD-B8ED-B7F9C2ADAE15

Figs 11–17 , 18–25 , 26–36 , 37–41 , 42–47 , 48–51 , 52–55

###### Type material.

Brazil, Pernambuco State, Catimbau National Park: Tupanatinga, Estrada do Gado [Cattle Road], 08°29'11.8"S, 37°20'25.5"W, 663 m alt., 19.iii.19, light trap, R. Carrenho leg., ***holotype***, male, 1 male paratype; light trap with white cloth, J.M.S. Rodrigues leg., 1 male paratype (CZMA); Buíque, ICMBio grounds, 08°33'54.9"S, 37°14'20.2"W, 730 m alt., 17.iii.19, light trap with white cloth, J.M.S. Rodrigues leg., 2 male paratypes (CTJMSB), 1 male paratype (CEIOC).

###### Diagnosis.

*Raceldaottoi* sp. nov. can be separated from other species of the genus by the combination of characters presented in the key below. *Raceldaottoi* sp. nov. shares similarities in coloration with *R.robusta* and *R.aberlenci* such as possessing both the fore lobe of pronotum and the abdomen, including the connexivum, mostly pale, while in the other species of the genus, these parts are mostly dark or at least, in case of the connexivum, it has well-defined dark markings on most segments. However, *R.ottoi* sp. nov. can be separated from *R.robusta* and *R.aberlenci* based on the coloration of the head and the legs, which are mostly blackish and with larger dark markings on femora and tibiae, respectively, in the former species, while in the latter two species, the head is completely pale, and the femora and tibiae are almost completely pale with the apices of femora and extremities of tibiae variably faintly dark marked or not marked. Additionally, while the longitudinal sulcus of pronotum is continuous on the two lobes in *R.robusta* and *R.aberlenci*, it is interrupted at the level of transverse sulcus in *R.ottoi* sp. nov.

###### Description.

**Male.** Figs 11–55. Measurements are given in Table 1.

**Table 1.** Measurements (in mm) of type specimens (*N* = 6) of *Raceldaottoi* sp. nov.

	Holotype	Mean	SD	Minimum	Maximum
Length to tip of abdomen	9.0	9.6	0.51	9.0	10.5
Length to tip of hemelytra (*N* = 5)^1^	9.2	9.73	0.56	9.2	10.6
Head length excluding neck	1.6	1.56	0.05	1.5	1.6
Head width across eyes	1.4	1.48	0.07	1.4	1.6
Synthlipsis	0.6	0.58	0.04	0.5	0.6
Eye width	0.4	0.41	0.04	0.4	0.5
Ocellar tubercle width	0.6	0.6	0.0	0.6	0.6
Ocellus width	0.2	0.24	0.04	0.2	0.3
Scape length	1.4	1.5	0.06	1.4	1.6
Pedicel length	1.6	1.67	0.10	1.6	1.8
Basiflagellomere I length	0.7	0.73	0.05	0.7	0.8
Basiflagellomere II length	0.4	0.47	0.05	0.4	0.5
Distiflagellomere I length (*N* = 5)^2^	0.25	0.27	0.02	0.25	0.3
Distiflagellomere II length (*N* = 5)^2^	0.2	0.2	0.0	0.2	0.2
Distiflagellomere III length (*N* = 5)^2^	0.15	0.19	0.02	0.15	0.2
Distiflagellomere IV length (*N* = 5)^2^	0.25	0.31	0.02	0.25	0.4
Labial segment II length	0.9	1.03	0.10	0.9	1.2
Labial segment III length	0.6	0.58	0.04	0.5	0.6
Labial segment IV length	0.3	0.41	0.09	0.3	0.5
Fore lobe of pronotum length	0.6	0.61	0.04	0.6	0.7
Fore lobe of pronotum max. width	2.0	2.0	0.0	2.0	2.0
Hind lobe of pronotum length	1.2	1.18	0.04	1.1	1.2
Hind lobe of pronotum max. width	2.9	2.95	0.05	2.9	3.0
Fore femur length	1.8	1.88	0.09	1.8	2.0
Fore tibia length	1.9	1.9	0.0	1.9	1.9
Fore tarsus length	0.7	0.7	0.0	0.7	0.7
Middle femur length	1.8	1.81	0.04	1.8	1.9
Middle tibia length	1.9	1.8	0.08	1.7	1.9
Middle tarsus length	0.7	0.68	0.04	0.6	0.7
Hind femur length	2.9	2.96	0.05	2.9	3.0
Hind tibia length	3.1	3.03	0.10	2.9	3.2
Hind tarsus length	1.0	1.01	0.04	1.0	1.1
Abdomen length*	5.4	5.51	0.17	5.3	5.7
Abdomen maximum width	3.3	3.63	0.20	3.3	3.9

^1, 2^ Distal portion of hemelytra and distiflagellomeres absent in one specimen. * Measured on ventral view, at midline, from anterior margin of sternite II to posterior border of last segment.

**Figures 18–25.**
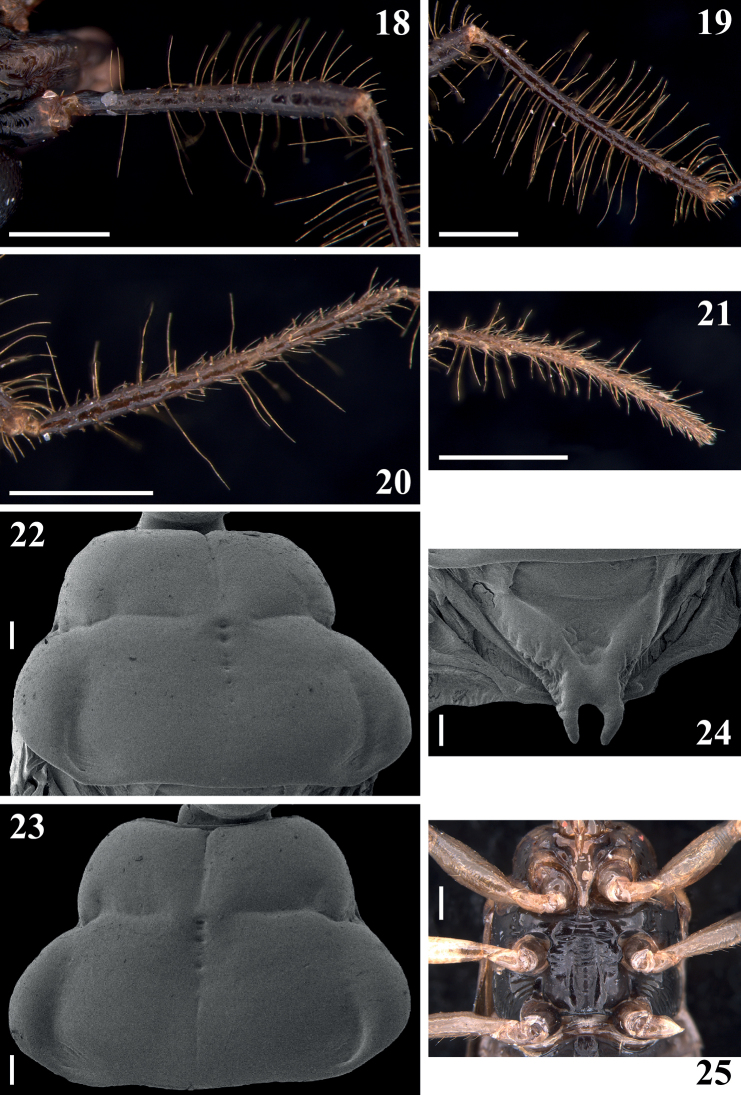
*Raceldaottoi* Oliveira & Gil-Santana, sp. nov. **18–24** dorsal view **18–21** antennal segments **18** scape and basal portion of pedicel **19** apical portion of scape and pedicel **20** basiflagellomeres **21** distiflagellomeres **22, 23** pronotum, different paratypes **24** scutellum **25** thorax, ventral view. Scale bars: 0.5 mm (**18–21, 25**); 0.2 mm (**22–24**).

**Figures 26–36.**
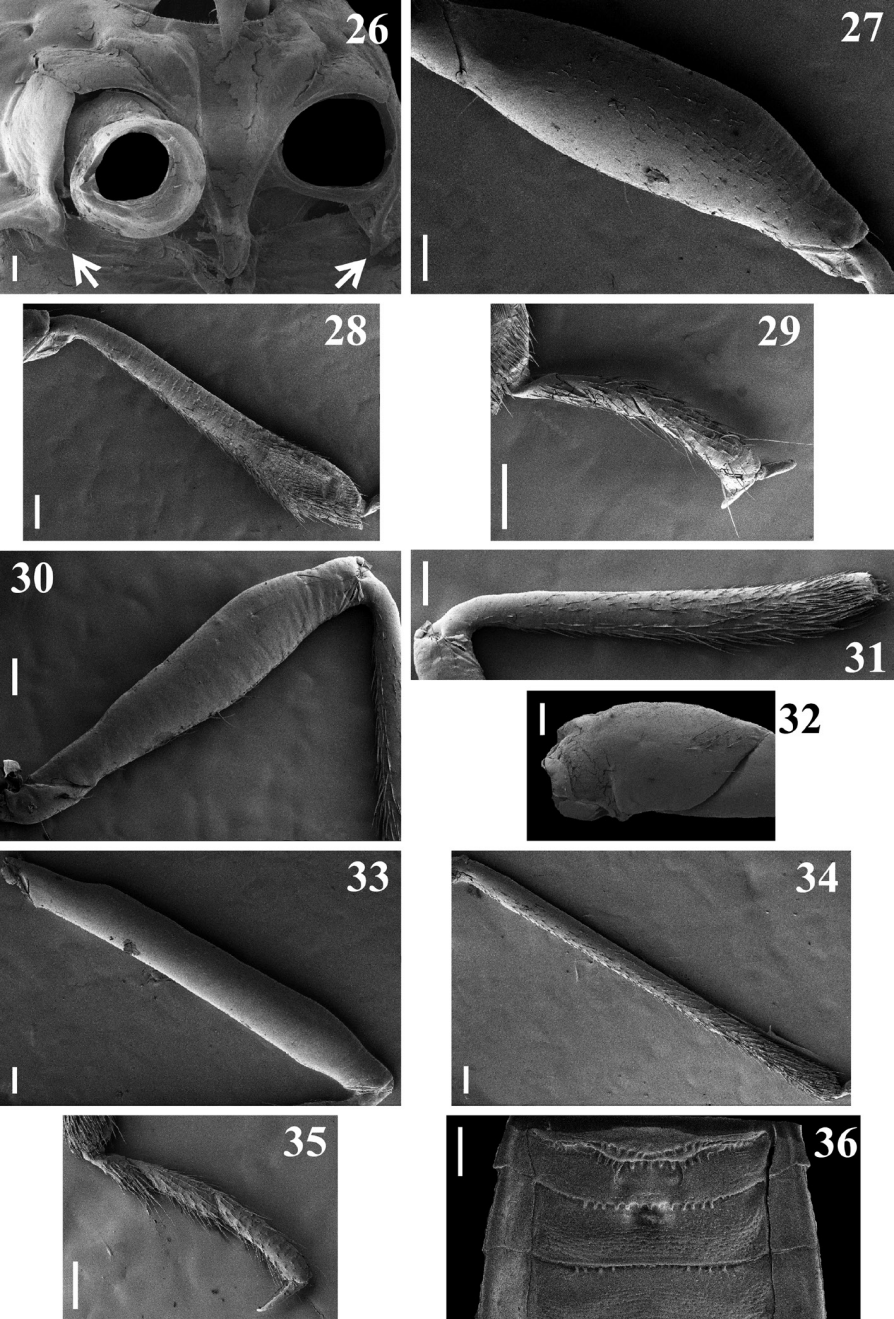
*Raceldaottoi* Oliveira & Gil-Santana, sp. nov. **26** prothorax, ventral view, arrows point to posteroventral elongate processes of propleura **27–29** fore leg **27, 28** lateral view, inner surface **27** femur **28** tibia **29** tarsus, dorsal view **30–35** lateral view, inner surface **30, 31** middle leg **30** trochanter, femur and basal half of ventral surface of tibia **31** tibia **32–35** hind leg **32** trochanter **33** femur **34** tibia **35** tarsus **36** abdomen, segments I–IV, except distal portion of the latter, dorsal view. Scale bars: 0.5 mm (**36**); 0.2 mm (**27–31, 33–35**); 0.1 mm (**26, 32**).

**Figures 37–41.**
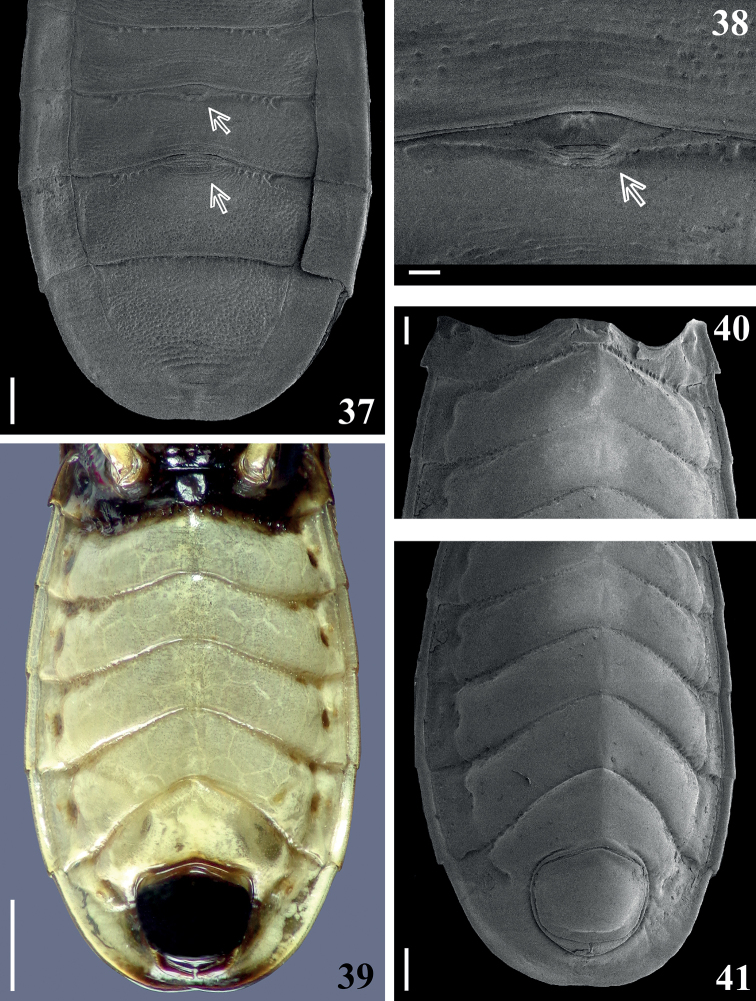
*Raceldaottoi* Oliveira & Gil-Santana, sp. nov., abdomen. **37, 38** dorsal view **37** distal portion of segment III and segments IV–VII, arrows point to the **dag** on tergites V and VI **38 dag** on basal portion of tergite V (pointed by an arrow) (**dag**: scar of dorsal abdominal gland opening) **39–41** ventral view **40** segments II–III, IV, except laterodistal portion, and midanterior portion of segment V **41** segments III (except basal portion), IV–VII. Scale bars: 1.0 mm (**39**); 0.5 mm (**37, 41**); 0.3 mm (**40**); 0.1 mm (**38**).

***Coloration***: general coloration pale to pale yellowish to orange with darkened to brownish or blackish portions or markings (Figs 11, 12, 25, 39). ***Head***, including neck, mostly blackish (Figs 11, 12); pale whitish to pale yellowish ventrally between the level of inner margin of eyes; a thin reddish line around ocelli, sometimes partially interrupted as in the holotype; a somewhat paler medial marking behind ocellar tubercle and on neck, variable in size, both present in the holotype and alternatively absent in some of the paratypes; base of neck paler in variable extension or completely dark as in the holotype; close to base of labium, on pale whitish ventral area, a small blackish marking as a thin transverse line or as a pair of separate markings; antennal segments (Figs 16–21) mostly dark with basal portion of scape and intersegmental joints pale; scape, pedicel and basiflagellomeres blackish to brownish black; distiflagellomeres somewhat paler, brownish; labrum paler on its distal half; labium pale to pale yellowish with apex of first visible segment darkened and faintly, irregularly marked with variably darkened portions such as a basoventral marking on segment II (first visible) and lateral and dorsal portions of segments III–IV; the latter entirely faintly darkened in one paratype. ***Thorax*** (Figs 11, 25): pronotum mostly pale orange, faintly darkened at collar, inferior portion of anterolateral angles, median portion of basal half of hind lobe and, in one paratype, medially to distal portion of humeral angles; scutellum blackish; propleura orange with a dark irregular marking extending above and/or anteriorly to fore supracoxal lobe, reaching prosternum at anterior portion, including their rounded processes; margins of prosternal process and its apex dark; meso- and metapleura and sterna mostly blackish; a small pale orange marking on median and distal portion of middle and hind supracoxal lobes, respectively. Legs: fore coxa whitish, pale with anterior surface variably darkened; middle and hind coxae from mostly pale to mostly darkened and paler only at distal margin; trochanters pale to pale yellowish; femora pale whitish, blackish on dorsal surface of fore (except at its base) and approximately distal third to distal fourth of middle and hind femora, respectively; fore femora also variably darkened at apex on lateral surfaces or even around segment; distal markings on middle and hind femora variably somewhat shorter on ventral surface; in one paratype blackish markings on middle and hind femora smaller, occupying only approximately distal fifth of segment; tibiae dark, brownish to blackish, with approximately their median portion variably paler, pale coloration occupying approximately median third on fore and middle tibiae and one-half on hind tibiae; pale coloration varying from almost as dark as dark extremities of segment, pale brownish to pale whitish; tarsi pale brownish. Hemelytra blackish; pale on base of dorsal surface, laterally, and on basal lateral portion; also slightly paler on apex of corium. ***Abdomen*** (Figs 11, 39): mostly pale whitish to pale yellowish; apex of connexival posterolateral angle of segment II somewhat darkened; in one paratype, connexival segment II completely darkened; sternite II mostly dark to blackish, except at posterolateral portion where it is pale in variable extension; intersternite furrow between between segments II–III and adjacent anterior portion of latter segment darker in one paratype (Fig. 39); midlateral subrounded shallow depressed areas on sternites III–VII faintly darkened; area posterior to genital capsule, on last sternite, darkened in most specimens, dark coloration varying in extension laterally; exposed portions of genital capsule and parameres dark to blackish. ***Structure***: Body integument mostly shiny. ***Head*** (Figs 11–21): shorter than pronotum (including neck); subtriangular in dorsal and lateral views. Vertex not elevated; minimum distance between eyes in dorsal view (synthlipsis) approximately 1.5–1.6 times longer than width of each eye. Antenna inserted proximal to midpoint between anterior margin of eyes and apex of head. Anteocular portion approximately twice as long as postocular portion (excluding neck); total length (excluding neck) of head longer than its maximum width across eyes; integument generally with coarsely transverse subparallel sulci or wrinkled; smooth on ocellar tubercle and neck. Clypeus moderately elongated, not elevated, rounded in lateral view, slightly wider at basal portion, its integument with transverse subparallel sulci. Antenna (Figs 16–21): scape somewhat curved and enlarged towards apex, slightly shorter than pedicel; the latter somewhat curved at midportion; flagellum slender, divided in pseudosegments, two basiflagellomeres and four distiflagellomeres; basiflagellomeres thinner than pedicel, first basiflagellomere longer than second; distiflagellomeres somewhat thinner than basiflagellomeres, first three subequal in length, last a little longer. Labium (Figs 14, 15) moderately thick, segment II (first visible) straight, somewhat thicker towards apex, approximately 1.5 times longer than the segment III, its apex approximately at level of anterior half of eyes in lateral view; segment III somewhat thicker; segment IV, shorter, tapering, reaching stridulatory sulcus approximately at its anterior third. Gula with lateral shallow longitudinal ridges, between which is a furrow narrower than labium, almost imperceptible in some individuals. Constriction between postocular portion and neck distinct (Fig. 13). Eyes large, prominent, subhemisphaerical in dorsal view, reniform in lateral view; transverse sulcus curved, reaching inner posterior angle of the eye (Figs 12, 13). Ocellar tubercle prominent, large, undivided, ocelli rounded, the distance between them somewhat closer than the diameter of each ocellus (Figs 12, 13). ***Thorax*** (Figs 11, 22–35): integument shiny; collar very thin; anterolateral angles pointed and small; fore lobe rounded on anterior and lateral margins, shorter and narrower than hind lobe; mid-longitudinal sulcus on fore lobe thin and narrow, ending somewhat above a median slightly elevated portion at median portion of transverse sulcus, with the remaining posterior part of the mid-longitudinal sulcus represented by few punctations, about half a dozen, two or three more anterior ones somewhat deeper and larger, followed by progressively smaller and shallower punctations towards posterior margin, shortly or not exceeding the distal half of hind lobe (Figs 22, 23), sometimes posterior to punctations, a very thin median longitudinal line ending short of posterior margin (Fig. 23); transverse furrow distinct, interrupted at median portion by the median elevated portion, sinuous, curved forward at lateral portion (Figs 22, 23), continuing laterally, on propleura, forming a somewhat curved lateral furrow, with short shallow ridges on anterior portion of its inferior margin, ending at posterior margin of propleura; posterolateral furrows of pronotum distinct; humeral angles rounded (Figs 11, 22, 23). Scutellum with a shallow median depression; scutellar prongs moderately short and curved, narrowly separated at base and convergent towards their apices (Fig. 24). Integument of pro- and mesopleura mostly smooth; faintly wrinkled by a few linear subparallel thin shallow linear impressions on supracoxal lobes; integument of metapleura with several linear subparallel irregular ridges, its superior margin thickened and curved. Supracoxal lobes of propleura somewhat prominent, those of meso- and metapleura not. Propleura with posteroventral elongate processes, apices acute, directed posteromedially, just posterior to laterodistal third of fore coxa, above lateral portion of anterior margins of mesosternum (Fig. 26). Anterior margin of mesopleura with a median small process, projecting anteriorly, rounded at apex which meets posterior margin of propleura. Prosternum wider on approximately anterior half, moderately large, prolonged between fore coxae, apex rounded, reaching mesosternum, with its median portion occupied by stridulitrum (Fig. 26). Mesosternum anteriorly to middle coxa mostly flattened and with smooth integument; on its median portion, just posterior to apex of process of prosternum, a small oval depression on midline, with elevated borders, below and laterally to which, a pair of subrounded small depressions; middle coxae bordered by slightly elevated margins anteriorly and medially. Between middle and hind coxae, a moderately elevated area with integument marked by few shallow transverse sulci and a pair of submedian shallow longitudinal ridges somewhat more elevated at distal half (Fig. 25). Fore coxae close, separated by a shorter distance than approximately half the width of each of them; middle and hind coxae distant from each other by a distance approximately equivalent to somewhat more than twice and approximately 1.7 times the width of each of them, respectively (Fig. 25). Fore and middle femora and tibiae subequally long; fore femora somewhat thickened, except at basal and distal portions (Fig. 27); middle femora thickened subapically (Fig. 30); hind femora and tibiae longer, slender, femora somewhat thickened subapically (Fig. 33). Tibiae straight, slightly longer than the correspondent femora; fore tibiae thicker at apex, in which the anterior margin is prominent and with a mesal comb (Fig. 28); middle and hind tibiae slightly thicker subapically and at apex, respectively (Figs 31, 34); spongy fossae on apices of fore and middle tibiae very small. All tarsi slender, three-segmented (Figs 29, 35). Hemelytra generally dull; moderately shiny on base of dorsal surface, laterally, and on lateral portion, basally (the same portions in which the coloration is pale) (Fig. 11). ***Abdomen*** (Figs 11, 36–41, 43): Tergite I narrow, carinulate on median portion of posterior margin (Fig. 36); other tergites shortly carinulate on posterior margin, except lateral portion, the ridges shorter on tergite IV, very faint on tergite VII (Figs 36, 37). Tergite II with its anteromedian portion bordered by a pair of longer curved longitudinal ridges (Fig. 36). Shallow and small punctations irregularly distributed on tergites III–VII; inner portion of respective connexival dorsal segment with few punctations (Figs 36, 37). Scars of dorsal abdominal glands openings (dag) on median anterior margins of tergites V and VI, that on the latter larger than that on tergite V; these tergites are carinulate only between the scars and lateral portion (Figs 37, 38). Connexivum with posterolateral angles of segments II–VI prominent, that of segment II somewhat more than the others (Figs 11, 36, 39–41). Sternites with shiny and generally smooth integument (Fig. 39); sternite II narrower than following segments, its median portion elevated (Figs 39, 40); sternites II and III separated by shallow canaliculae, which are absent at lateral portions; other intersternite furrows with small, shallow punctations, absent in midline, sparser or absent towards lateral portions and less numerous on last interstenite furrow; sternites III–VII with smooth integument and midlateral subrounded shallow flat depressions, anterior portion of the latter close and medial to the respective spiracles (Figs 39–41). Spiracles round and small. Posterior margin of segment VII slightly curved anteriorly at its midportion (Fig. 39). Segment VIII not visible externally, sclerotized on ventral portion, which is translucent; segment becomes wider towards posterior margin; both basal and distal margins of ventral portion curved, the former more than the latter and more sclerotized than remaining part of segment (Fig. 43); dorsal portion membranous and narrower; spiracles on dorsal margin of ventral portion. ***Vestiture***: integument generally mostly glabrous. ***Head***: some long moderately curved pale setae scattered on the anterior and lateral portions of base of first visible labial segment; several long, erect, pale setae on apical portion of second visible labial segment and scattered on last labial segment. Antenna (Figs 16–21): scape with some and pedicel with few short, oblique, thin setae, and both segments covered by long pubescence formed by numerous long, erect, stout, pale setae, approximately twice as long as width of scape and 4–5 times as long as width of pedicel, except at distal portion, where these setae are shorter; flagellomeres covered by very numerous short, oblique, curved, thin pale setae, forming a short dense pubescence, except on approximately basal half of first basiflagellomere, where setae are less numerous; flagellomeres also covered by long stout, erect pale setae; those on first basiflagellomere almost as long and numerous than those of pedicel, becoming progressively shorter and less numerous on following flagellomeres. ***Thorax***: pronotum with a tuft of golden stout setae on inner margin of posterior border of pronotum beside lateral margin of scutellar base. Borders of posterior prolongation of prosternum with thin, longer, dark golden setae; some scattered pale thin setae on postacetabular area of prosternum laterally to the prosternal process. Legs: coxa with some stout, curved, pale or somewhat darkened, thin setae on apical margin; trochanters and ventral surface of femora with several curved, stout and curved pale, thin setae (Figs 30, 32); fore trochanter and fore femur with also at least one thinner, longer subbasal seta; some scattered similar long setae on dorsal, lateral and ventral surfaces of femora, on fore femora some rows of curved, thin, pale, shorter setae on dorsal surface (Fig. 27); tibiae glabrous at approximately basal third to two-thirds of dorsal surface, with a mid-ventral fringe of short, straight, somewhat stouter, pale brownish setae; at approximately distal third, all tibiae generally covered by pale brownish to darkened setae, which become somewhat more numerous towards apex, where they are longer on ventral and lateral surfaces (Figs 28, 31, 34); tarsi covered with numerous yellowish and golden setae, which are longer on ventral surface (Figs 29, 35).

**Figures 42–47.**
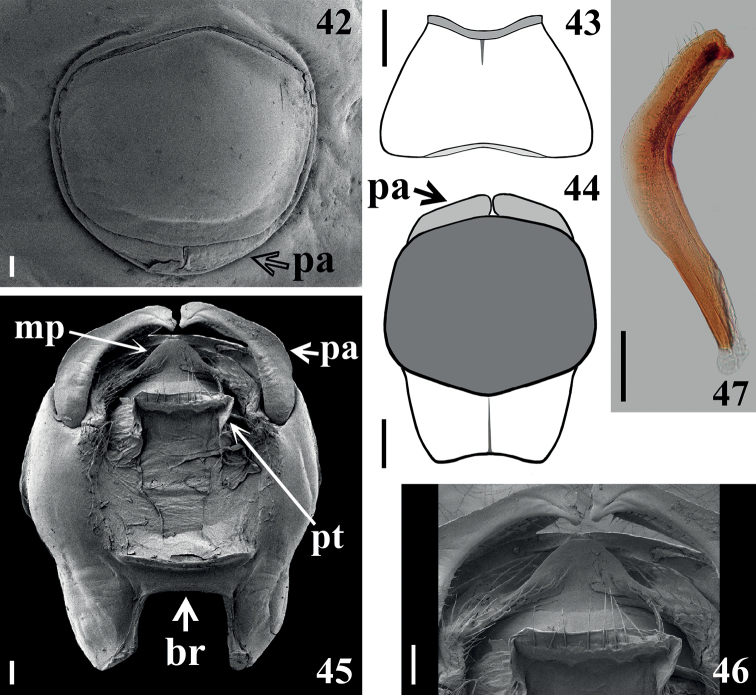
*Raceldaottoi* Oliveira & Gil-Santana, sp. nov., male genitalia. **42** genital capsule “in situ”, ventral view **43, 44** schematic outline, ventral view **43** abdominal segment VIII, **44, 45** pygophore **45, 46** parameres slightly moved apart, dorsal view **46** apical portions of parameres and proctiger and medial process of pygophore **47** right paramere. Abbreviations: **br**: transverse bridge; **mp**: medial process of pygophore; **pa**: paramere; **pt**: proctiger Scale bars: 0.3 mm (**43, 44**); 0.2 mm (**47**); 0.1 mm (**42, 45, 46**).

**Figures 48–51.**
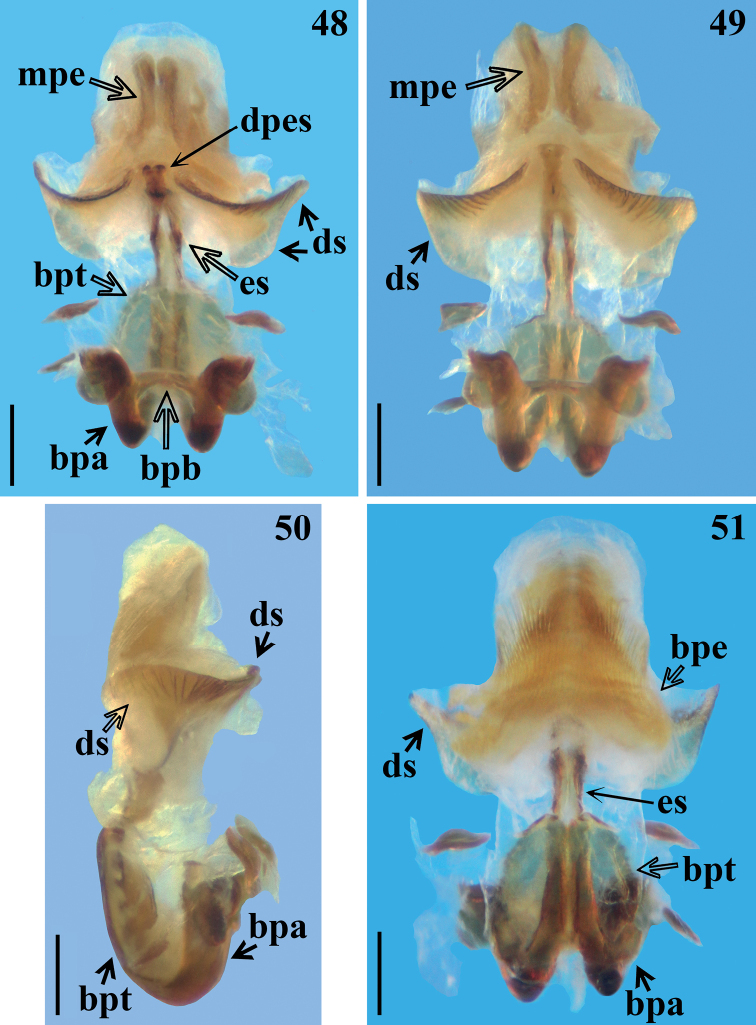
*Raceldaottoi* Oliveira & Gil-Santana, sp. nov., male genitalia, phallus. **48, 49** dorsal view **50** lateral view **51** ventral view. Abbreviations: **bpa**: basal plate arm; **bpb**: basal plate bridge; **bpe**: basal process of endosoma; **bpt**: basal plate extension; **dpes**: dorsal phallothecal sclerite-endosomal struts fusion; **ds**: dorsal phallothecal sclerite; **es**: endosomal struts; **mpe**: median process of endosoma. Scale bars: 0.3 mm (**48–51**).

**Figures 52–55.**
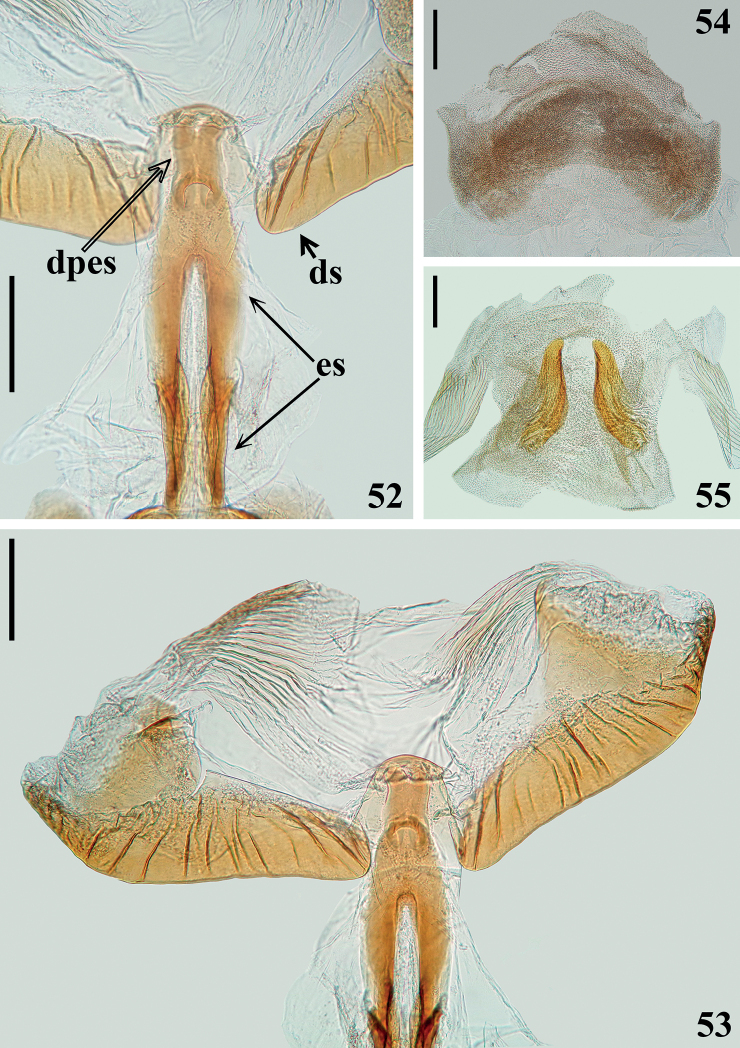
*Raceldaottoi* Oliveira & Gil-Santana, sp. nov., male genitalia, dorsal view. **52** endosomal struts (**es**), dorsal phallothecal sclerite-endosomal struts fusion (**dpes**) and median portions of distal thickened margin of dorsal phallothecal sclerite (**ds**) **53** distal half of endosomal struts, dorsal phallothecal sclerite-endosomal struts fusion and dorsal phallothecal sclerite **54** basal process of endosoma **55** median process of endosoma. Scale bars: 0.2 mm (**52–55**).

***Male genitalia*.** Pygophore, in ventral and lateral views: exposed portion of pygophore subpentagonal (Figs 39, 41, 42, 44) and rounded, respectively, integument smooth and shiny; only pigmented (blackish) in the exposed portion (Figs 39, 44); in dorsal view (Fig. 45): between anterior and posterior genital openings, a moderately broad, slightly sclerotized dorsal (transverse) straight bridge (br); membranous areas of posterior genital opening smooth; proctiger (pt) subsquared, posterior margin almost straight, with a subapical single row of few long straight setae (Figs 45, 46). Medial process of pygophore (mp) sclerotized, subrounded, apical margin almost straight (Figs 45, 46). Parameres (pa) mildly exposed when genital capsule is in situ (Fig. 42) their apices in contact in resting position (Figs 42, 44); symmetrical, elongated, curved at approximately middle third, where they are somewhat larger; apex truncated, with a rounded subapical tooth in inferior margin; mostly glabrous, with some rows of long setae on inner surface, a few subapical short somewhat stout setae on upper surface and a row of somewhat curved very short setae above the subapical tooth (Figs 46–47). Phallus: articulatory apparatus with basal plate extension (bpt) enlarged, longer than basal plate, the latter with moderately short and curved basal plate arms (bpa), connected by a narrow basal plate bridge (bpb) (Figs 48–51). Dorsal phallothecal sclerite (ds) symmetrical, enlarged to apex; distal margin thickened and more sclerotized, with several linear grooves interrupted at midportion beside apex of struts and dorsal phallothecal sclerite–endosomal struts fusion (dpes) (Figs 48, 49, 52, 53). Endosomal struts (es) formed by a pair of parallel arms, somewhat thinner at basal third, slightly enlarging toward apex, where they converge and become largely united, being continuous with dorsal phallothecal sclerite–endosomal struts fusion (dpes) (Fig. 52). Endosoma wall longitudinally striated on basal portion, ventrally (Fig. 51), smooth basally, and mostly very densely minutely, spiny towards apical portion (spines minute). Two processes of endosoma: a wide, arcuate, basal process (bpe) formed by diffuse thickening (Figs 51, 54) and a median process (mpe) at apical portion (Figs 48, 49). Median process formed by a pair of elongate, flat, somewhat curved, moderately sclerotized plates (Fig. 55).

###### Distribution.

Brazil, state of Pernambuco.

###### Etymology.

The new species is named in honor to Otto Pompeu Fusco de Oliveira, the beloved son of the junior author (JO).

###### Comments.

The inclusion of *R.ottoi* sp. nov. in *Racelda* is in accordance with the characteristics assigned to the species of this genus by Dougherty (1995), Carpintero and Maldonado (1996), and Forero (2004). *Raceldaottoi* sp. nov. differs from the other species of the genus by the combination of characteristics stated in the key for males of *Racelda*, presented below and from the species to which it seems closest, *R.aberlenci* and *R.robusta*, by the set of features commented on in its diagnosis.

The antennae of most of the New World Ectrichodiinae males are pubescent on all segments with short setae but which are more abundant on the distal segments (Dougherty 1995). Dougherty (1995) also stated that in *Racelda* the antennae of males have long and short pubescence on all segments. In *R.ottoi* sp. nov., the setae of the long pubescence are longer and formed with more numerous elements on the scape and pedicel (Figs 16–19), becoming progressively shorter and less numerous on the following segments (flagellomeres), while the short pubescence is almost absent on the scape and pedicel and become progressively more numerous to very dense on the flagellomeres (Figs 16, 17, 20, 21). Yet, the males of the new species present large eyes and well-developed ocelli, and they are macropterous (Figs 11–13). Although all these characteristics are similar to those presented by males of Ectrichodiinae and *Racelda* in contrast with conspecific females, which have very reduced eyes and ocelli and are apterous (Dougherty 1995; Carpintero and Maldonado 1996), because no females of *R.ottoi* sp. nov. were found, the extent of sexual dimorphism, including possible differences in coloration, will only be known if more specimens of both sexes become available in the future for examination.

**Figures 56–62.**
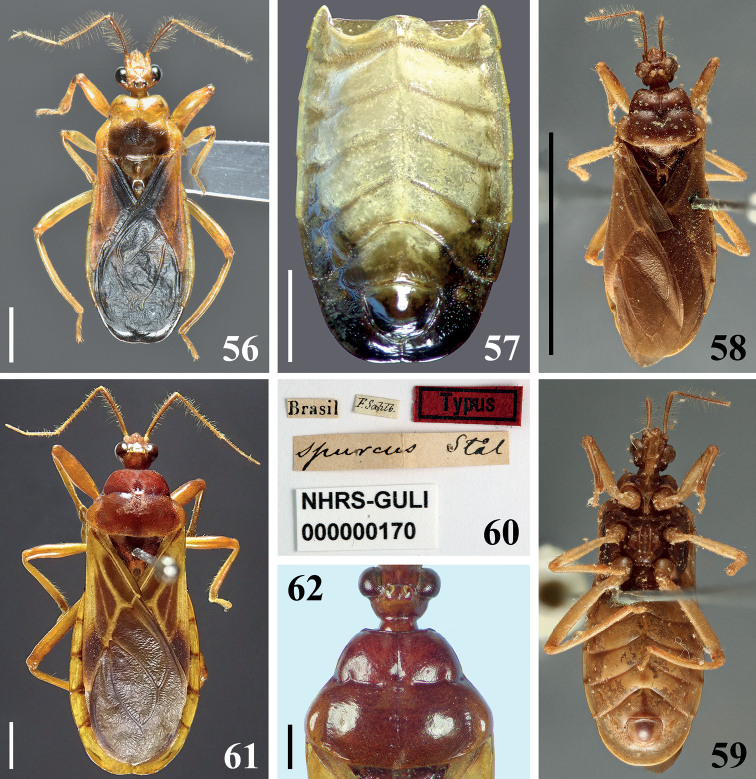
**56, 57***Raceldarobusta* Bérenger & Gil-Santana, 2005, male from French Guiana **56** dorsal view **57** abdomen, ventral view **58–62***Raceldaspurca* (Stål, 1860) **58–60** male syntype deposited in NHRS, catalog number NHRS-GULI000000170, photographed by Gunvi Lindberg, © 2020 Naturhistoriska riksmuseet. Made available by the Swedish Museum of Natural History under Creative Commons Attribution 4.0 International Public License, CC-BY 4.0, https://creativecommons.org/licenses/by/4.0/legalcode**58** dorsal view, scale bar modified from original **59** ventral view **60** labels **61, 62** male from Brazil, dorsal view **62** distal portion of head and pronotum. Scale bars: 10.0 mm (**58**); 2.0 mm (**57, 61**); 1.0 mm (**56, 62**).

Interestingly, the mid-longitudinal sulcus on hind lobe of pronotum was represented by a few punctations, about half a dozen, and the two or three more anterior ones were somewhat deeper and larger, scarcely or not exceeding the distal half of the hind lobe (Fig. 11), while after obtaining SEM images (Figs 22, 23), it was possible to record that sometimes, posterior to the punctations, there is also a very thin median longitudinal line ending short of posterior margin (Fig. 23). Therefore, there is an intraspecific variation in the extension and characteristics of the mid-longitudinal sulcus on the hind lobe in *R.ottoi* sp. nov.

##### ﻿ Racelda robusta

Taxon classificationAnimaliaHemipteraReduviidae

Bérenger & Gil-Santana, 2005

9C97E36D-6989-5535-A122-EFB36E5FC7A7

Figs 56 , 57

###### Material examined.

French Guiana, Bélizon, xi.2001, H. Gaspard leg., 2 males (MNRJ).

*Raceldarobusta* was described based on a male from French Guiana (Bérenger and Gil-Santana 2005). Gil-Santana et al. (2013) recorded the species from Brazil (Amazonian region). They observed that while the holotype had the center of hind lobe of pronotum entirely blackish, the male from Brazil had a less extensive blackish coloration, with a pale portion below the transverse sulcus. In the males from French Guiana examined here, the area below the tranverse sulcus is also pale to some extent, except at medially (Fig. 56), suggesting intraspecific rather than geographic variation in the size of this blackish marking.

##### ﻿ Racelda spurca

Taxon classificationAnimaliaHemipteraReduviidae

(Stål, 1860)

89150E8A-B08B-56D1-A587-11D4AA9F350D

Figs 58–62

###### Material examined.

Brazil, São Paulo State: *RACELDA* / *spurca* (Stäl [handwritten] / J.C.M. Carvalho. det. 1991 [printed; except two latter numbers, which were handwritten] // [handwritten label]: 20.XI.1955 / Barueri / K. Lenko leg., 1 male (MNRJ).

*Raceldaspurca* was described based on an unspecified number of male specimens from Rio de Janeiro, Brazil. Stål (1860, 1872) cited “Mus. Holm.” (NHRS) as the depository of the type specimen(s). Currently, there is only one type specimen of *R.spurca* deposited there (G. Lindberg pers. comm.) (Figs 58–60). The possibility that the species was described based on more than one specimen cannot be excluded and, following Art. 73.2 and the Recommendation 73F of the ICZN, this specimen is therefore considered as a syntype.

Some characteristics of a non-type male specimen examined here (Figs 61, 62) are noteworthy, such as the presence of pale veins on the coria of hemelytra (Fig. 61) and the longitudinal sulcus of the pronotum, which is clearly interrupted at the level of transverse sulcus (Fig. 62) and not continuous along the two lobes.

## ﻿Discussion

Considering the fact that the females of most species of *Racelda* are unknown, it is possible that *Raceldamonstrosa*, described based only on a female, may be conspecific with one of the species of *Racelda* in which only the males are known so far. However, this possibility seems unlikely for *R.aberlenci*, *R.ottoi* sp. nov., and *R.robusta* because of the large differences in general coloration and size. While *R.monstrosa* is generally dark and larger (total length to the tip of abdomen 20 mm) (Carpintero 1980), the other species have several pale portions and are quite smaller, with the following total length: *R.aberlenci* (11 mm), *R.ottoi* sp. nov. (9–10.5 mm), and *R.robusta* (13.5 mm) (Bérenger and Gil-Santana 2005; this work).

It is noteworthy that some of the diagnostic characteristics of *Racelda* stated by previous authors were not present in some species or specimens studied here. The longitudinal sulcus has been described as extending along both lobes of pronotum, i.e., as continuous on the two lobes (Carpintero and Maldonado 1996; Forero 2004), but it may be interrupted at the level of transverse sulcus (e.g., in *R.ottoi* sp. nov. (Figs 22, 23) and *R.spurca* (Fig. 62)). Yet, in *R.ottoi* sp. nov., SEM images of two different specimens showed intraspecific variation, in which, posterior to the punctations on anterior portion of hind lobe, sometimes there is also a very thin median longitudinal line ending short of posterior margin (Fig. 23). Therefore, it is plausible to consider that in other species such variation in the extension of longitudinal sulcus on hind lobe may occur; more specimens need investigation to determine this. In this case, it is more appropriate to generally consider the mid-longitudinal sulcus simply as being well developed anteriorly and obsolete posteriorly as stated by Dougherty (1995). On the other hand, Dougherty (1995) in her key to Ectrichodiinae genera stated that in *Racelda* the anterolateral corners of pronotum would always be squared. However, although in many species they really seem roughly squared (e.g., *R.aberlenci*, *R.moerens*, *R.robusta*, and *R.spurca*) (Figs 1, 9, 56, 58), in *R.alternans*, the type species of the genus, they are definitively rounded (Figs 3, 6). Therefore, although this characteristic might be maintained among features presented by species of *Racelda*, it must not be posited as the deciding factor for including specimens/species in *Racelda* as stated in the Dougherty’s key. It may be necessary in the future to redefine the genus *Racelda* to accommodate these variations.

### ﻿Key to the species of *Racelda* Signoret, 1863 (males only)

**Table:** 

1	Connexivum with alternating pale and dark portions on segments III–VI	**2**
–	Connexivum without alternating pale and dark portions	**3**
2	Fore femora only slightly thickened (Figs 4, 7); connexival dark markings on segments III–VI, occupying approximately the distal half to distal third of these segments (Figs 4, 6, 7)	***alternans* Signoret, 1863**
–	Fore femora clearly thickened (Fig. 61); connexival dark markings on segments III–VI occupying only distal margin of these segments (Figs 58, 61)	***spurca* (Stål, 1860)**
3	Pronotum, connexivum and sternites mostly darkened (Fig. 9)	***moerens* Breddin, 1898**
–	Most part of pronotum (or at least its fore lobe and humeral angles), connexivum and sternites mostly or completely pale	**4**
4	Coria of hemelytra mostly dark yellow to orange (Fig. 56); segments VI–VII of connexivum, lateral portions of sternites V–VI and sternite VII almost or completely dark to blackish (Fig. 57)	***robusta* Bérenger & Gil-Santana, 2005**
–	Coria of hemelytra mostly dark to blackish, with only the basolateral portion pale and sometimes the apex faintly paler; connexival segments III–VII and sternites III–VI pale (Figs 1, 2, 11, 39); sternite VII sometimes somewhat darkened only on the portion posterior to genital capsule (Fig. 39), otherwise completely pale too	**5**
5	Head completely pale (Fig. 1); pronotum: mid-longitudinal sulcus of pronotum continuous on two lobes; fore lobe pale, hind lobe mostly dark, with portions lateral to postero-lateral furrows pale or with faint dark markings (Fig. 1); legs mostly pale with femoro-tibial joints and apices of tibiae variably faintly darkened (Fig. 1); sternite II pale (Fig. 2)	***aberlenci* Bérenger & Gil-Santana, 2005**
–	Head mostly blackish (Figs 11, 12), pale to whitish ventrally, between level of inner portion of eyes; pronotum: mid-longitudinal sulcus on fore lobe interrupted somewhat above a median elevated portion of transverse sulcus (Figs 22, 23); mostly orange, faintly darkened at median portion of basal half of hind lobe (Fig. 11); legs: fore femora extensively blackish dorsally, middle and hind femora blackish on their distal half and distal third, respectively; tibiae darkened with their median portion paler (Fig. 11); sternite II almost completely dark (Fig. 39)	***ottoi* Oliveira & Gil-Santana, sp. nov.**

## Supplementary Material

XML Treatment for Racelda aberlenci

XML Treatment for Racelda alternans

XML Treatment for Racelda moerens

XML Treatment for Racelda ottoi

XML Treatment for Racelda robusta

XML Treatment for Racelda spurca
